# Body mass index on perinatal depression: A critical viewpoint

**DOI:** 10.1192/j.eurpsy.2023.2447

**Published:** 2023-09-29

**Authors:** David F. Lo, Jaylyn Thompson

**Affiliations:** Department of Medicine, Rowan University School of Osteopathic Medicine, Stratford, NJ, USA

**Keywords:** body mass index, maternal well-being, perinatal depression, pregnancy outcomes

## Abstract

I am pleased to submit a viewpoint titled “Body mass index on perinatal depression: A critical viewpoint” in response to an article by Ventriglio et al. titled, “The impact of body mass index on the pregnancy outcomes and risk of perinatal depression: Findings from a multicenter Italian study” for consideration for publication in your journal.

## Editor-in-Chief, European Psychiatry

To the Editor-in-Chief,

I am pleased to submit a viewpoint titled “Body mass index on perinatal depression: A critical viewpoint” in response to an article by Ventriglio et al. titled, “The impact of body mass index on the pregnancy outcomes and risk of perinatal depression: Findings from a multicenter Italian study” for consideration for publication in your journal.

This article is not under consideration for publication elsewhere and will not be until a decision is made. All authors have contributed to and approved the final manuscript with all authors reporting no competing interests. Regarding IRB, this letter was exempt from the Rowan Institutional Review Board approval.

David F. Lo will be the corresponding author for any queries regarding this article and has full personal access to all parts of the writing process and takes full and final responsibility for the letter. We hope this letter will be valuable to your readers because it helps the authors to identify the areas for further research and investigation.

Thank you in advance for your consideration of the letter. I look forward to your comments.

Sincerely,

David F. Lo, MBS

Department of Medicine, Rowan School of Osteopathic Medicine

Address: 1 Medical Center Dr, Stratford, NJ 08084, USA

Email: Lodavi26@rowan.edu

Phone: (973) 996-8669

Body mass index (BMI) and perinatal depression (PD) are two crucial factors that intersect during pregnancy, influencing maternal well-being and pregnancy outcomes. Only recently have these factors been studied by Ventriglio et al. in a paper titled, “The impact of body mass index on the pregnancy outcomes and risk of perinatal depression: Findings from a multicenter Italian study” [1]. In this viewpoint paper, we critically examine the study’s findings and share insights into some of the current literature on this topic. This paper sheds light on the potential implications of BMI on both mental health and physical well-being during the perinatal period, opening avenues for deeper exploration and evidence-based interventions.

The multicenter Italian study contributes valuable insights into the association between BMI and perinatal depression risk and explores this relationship in a large multicenter sample of 1,611 pregnant women. Using standardized measures, such as the Edinburgh Postnatal Depression Scale (EPDS) and assessments of neuroticism, resilience, and quality of life, enhances the validity and comparability of the findings. The identification of associations between higher BMI, increased risk of perinatal depression, and poorer psychological well-being underscores the importance of addressing mental health issues in pregnant women with elevated body weight. The study also highlights the potential impact of BMI on pregnancy outcomes, linking higher BMI to increase medical treatments, physical comorbidities, and complications during pregnancy. This aspect of the research underscores the need for proactive monitoring and management of weight-related concerns during pregnancy to mitigate potential adverse effects on maternal and fetal health [1].

While the study identifies associations between BMI and perinatal depression risk and pregnancy outcomes, it does not establish causality. Other unmeasured confounding factors, such as socioeconomic status, social support, genetic predisposition, and underlying mental health conditions might influence these relationships [2]. Future studies can employ longitudinal designs and consider these potential confounders to establish causal relationships. The temporal relationship between BMI changes and the onset of perinatal depression is an essential aspect that requires further investigation [3]. The existing study provides a snapshot of BMI and perinatal depression at specific time points during pregnancy (third trimester) and after childbirth. However, it does not offer a detailed understanding of how BMI fluctuations and the development of perinatal depression might be related over time. For instance, a recent paper published by Taniguchi et al. in *Scientific Reports* discussed how weight-loss behaviors before pregnancy were associated with an increased risk of postpartum depression [4]. This poses exciting aspects to the relationship between them, whether perinatal or postpartum depression is due to starting weight/BMI or changes in this measurement.

The study also indicated that psycho-educational interventions could potentially assist in addressing physical and emotional concerns during pregnancy. However, it lacks specific information regarding the nature of these interventions and their demonstrated effectiveness. Future research can focus on identifying evidence-based psycho-educational interventions tailored to pregnant women with higher BMI, which have proven effective in reducing perinatal depression risk and improving pregnancy outcomes [5]. For instance, Steardo et al. published a paper on various psychoeducational interventions for perinatal depression such as individual and family assessment, communication and problem-solving skills, early warning signs, and management of suicidal behaviors. The authors also provided a framework for designing novel interventions to effectively treat such conditions [6].

Overall, Ventriglio et al. have made a significant and commendable contribution to our understanding of the intricate relationship between BMI, PD risk, and pregnancy outcomes. Their study represents a pivotal step forward in shedding light on a critical yet often overlooked aspect of maternal and child health. By examining the associations between these variables, the authors have not only provided valuable insights but have also underlined the imperative for comprehensive mental health screening and support strategies targeted specifically at pregnant women with higher BMI. Future studies should explore temporal aspects, consider confounding factors, and explore effective psycho-educational interventions to support the mental health and well-being of pregnant women.
